# Exploring the synergy between fungal CE15 glucuronoyl esterases and xylanases for lignocellulose saccharification

**DOI:** 10.1186/s13068-025-02639-0

**Published:** 2025-03-26

**Authors:** Christina Pentari, Constantinos Katsimpouras, Mireille Haon, Jean-Guy Berrin, Anastasia Zerva, Evangelos Topakas

**Affiliations:** 1 Industrial Biotechnology & Biocatalysis Group, School of Chemical Engineering, National Technical University of Athens, 9 Iroon Polytechniou Str., Zografou Campus, 15772 Athens, Greece; 2 Department of Chemical Engineering, Massachusetts Institute of Technology, 77 Massachusetts Avenue, Cambridge, 02139 MA USA; 3 INRAE, Aix Marseille Univ., BBF, Biodiversité et Biotechnologie Fongiques, 13009 Marseille, France; 4 Laboratory of Enzyme Technology, Department of Biotechnology, School of Applied Biology and Biotechnology, Agricultural University of Athens, 75 Iera Odos Street, 11855 Athens, Greece

**Keywords:** Lignocellulosic biomass, Beechwood, Corn bran, Synergy, Lignin–carbohydrate complexes, Xylanases, GH30 glucuronoxylanase/xylobiohydrolase

## Abstract

**Background:**

Lignin–carbohydrate complexes in lignocellulosic biomass act as a barrier to its biodegradation and biotechnological exploitation. Enzymatic dissociation between lignin and hemicellulose is a key process that allows the efficient bioconversion of both polymers. Glucuronoyl esterases of the Carbohydrate Esterase 15 family target the ester linkages between the glucuronic acid of xylan and lignin moieties, assisting enzymatic biodegradation of lignocellulose.

**Results:**

In this study, two CE15 glucuronoyl esterases from the white-rot fungi *Artolenzites elegans* and *Trametes ljubarskyi* were heterologously expressed in *Pichia pastoris* and biochemically characterized on the model substrate D-glucuronic acid ester with cinnamyl alcohol and a variety of pretreated lignocellulosic biomasses. The pretreatment method was shown to be a determining factor in revealing both the activity of the esterases on lignocellulose and their synergistic relationships with other hemicellulases. *Ae*GE15 and *Tl*GE15 demonstrated activity on pretreated biomass with high hemicellulose and lignin content, increasing saccharification by 57 ± 1 μM and 61 ± 3 μM of xylose equivalents, respectively. Furthermore, the synergy between these CE15 esterases and three xylanases from distinct glycoside hydrolase families (GH10, GH11 and GH30) was investigated on pretreated lignocellulosic samples, highlighting beneficial enzymatic interplays. Pretreated birchwood degradation by *An*Xyn11 was increased from 6% to approximately 10% by the esterases, based on xylose equivalents of unsubstituted xylooligomers. The GEs also promoted the glucuronoxylanase specificity of *Tt*Xyn30A, leading up to three-times higher release in aldouronic acids. Finally, a synergistic effect between *Ae*GE15 and *Tm*Xyn10 was observed on pretreated corn bran, increasing xylose and xylotriose release by 27 ± 8% and 55 ± 15%, respectively.

**Conclusions:**

Both CE15 esterases promoted biomass saccharification by the xylanases, while there was a prominent effect on the GH30 glucuronoxylanase regarding the release of aldouronic acids. Overall, this study shed some light on the role of CE15 glucuronoyl esterases in the enzymatic biodegradation of plant biomass, particularly its (arabino)glucuronoxylan component, during cooperative activity with xylanases.

**Supplementary Information:**

The online version contains supplementary material available at 10.1186/s13068-025-02639-0.

## Background

Lignocellulosic biomass is the most abundant renewable carbon source on earth, derived mainly from agricultural and forest residues [51]. Consisting of a complex network of cellulose, hemicellulose and lignin, lignocellulose can be used as raw material for numerous biotechnological applications [63]. The lignin–carbohydrate complexes (LCCs), formed through covalent bonds between lignin and hemicellulosic polysaccharides, pose a severe barrier in biomass utilization [4]. Glucuronoxylan and (glucurono)arabinoxylan are typical heteroxylan types that participate in LCCs formation [63]. Part of the 4-*O*-methyl-glucuronoyl (MeGlcA) moieties of woody hemicellulose is esterified in LCCs [40], while arabinosyl residues participate in LCCs via esterifications to ferulic acid, which can be further ether-linked or C–C linked to lignin moieties [13].

Enzymatic disassociation of lignin from polysaccharides is a necessary step towards the exploitation of all biomass components. The ester bonds between the MeGlcA moiety of xylan and an alcohol part in LCCs can be degraded by glucuronoyl esterases (GEs) [7, 38, 48, 66], which belong to the carbohydrate esterase 15 (CE15) family (EC number 3.1.1.117) [14]. Since their discovery almost two decades ago, the elucidation of their catalytic mechanism and biological role has proven to be quite challenging tasks [15, 30, 53]. Despite their selectivity primarily for glucuronoyl esters, most GEs have no restrictions on the size and substitution pattern of the lignin and hemicellulosic part of their substrate, exhibiting a preference for bulkier structures [11, 12, 37].

Formerly, GE activity was studied on model substrates, involving GlcA-based esters with variable alcohol substituents representing the ‘lignin side’ of LCCs [27, 54, 56, 60]. To better simulate the proposed native substrate of GEs, native LCCs have been isolated from biomass [7, 12, 37]. As a result, activity of *Cu*GE (*Cerrena unicolor*), *Ps*GE (*Punctularia strigosozonata*), *Tt*GE (*Thielavia terrestris*), and *Afu*GE (*Armillaria fuscipes*) was verified on LCC-rich substrates from birch [37, 38]. Apart from hardwood, GEs were also found to act on LCCs from spruce and corn [37, 57], and other rare glucuronoxylan structures [34]. Even though GEs have been found to interact both with the hemicellulosic and the lignin part of the model substrates [37], further research is required to gain a better insight in their activity on native lignocellulose and investigate their potential in biomass valorization.

The widespread presence of GE-encoding genes in lignocellulolytic microorganisms suggests their importance for unlocking biomass cell wall recalcitrance [2, 31, 36,30]. However, their synergistic interactions with other hemicellulases during the cooperative degradation of lignocellulosic biomass have rarely been studied. To our knowledge, there are only a few studies focusing on the synergism between GEs and (hemi)cellulolytic preparations [2, 11, 48, 57, 64]. Therefore, their role in natural mechanisms of plant biomass deconstruction is still elusive. This work aims to address the synergistic relationships between GEs and xylanases of different glycoside hydrolase (GH) families on natural lignocellulosic biomass. For this reason, two fungal GEs from *Artolenzites elegans* and *Trametes ljubarskyi* (synonym *Pilatotrama ljubarskyi*), namely, *Ae*GE15 and *Tl*GE15, were heterologously expressed in *Pichia pastoris* and characterized. Their activity on recalcitrant glucuronoyl esters of pretreated biomass was verified. Finally, the effect of GEs on the hydrolysis of lignocellulosic biomass was investigated, in combination with xylanases with distinct specificities.

## Results and discussion

### GEs heterologous expression and biochemical characterization

The two fungal GEs studied herein belong to the CE15 family of CAZymes and were selected based on a previous study [42]. They were both identified using proteomics in the secretomes of the two basidiomycete strains, i.e., *A. elegans* (GenBank: KAI0768804) and *T. ljubarskyi* (GenBank: KAI0373448), when these fungal sparotrophs were grown on lignocellulosic biomass. Both CE15s were found among a very diverse set of CAZymes (cellulases, hemicellulases, pectinases). The *aege* gene contains 4 introns (58, 57, 59 and 49 bp) and encodes a protein of 471 amino acids, including a signal peptide of 21 amino acids. The *tlge* gene involves 4 introns (61, 52, 51 and 54 bp) and encodes a protein of 474 amino acids, including a signal peptide of 21 amino acids. Both GEs contain a carbohydrate-binding module of the CBM1 family on their N-terminal.

**Fig. 1:**
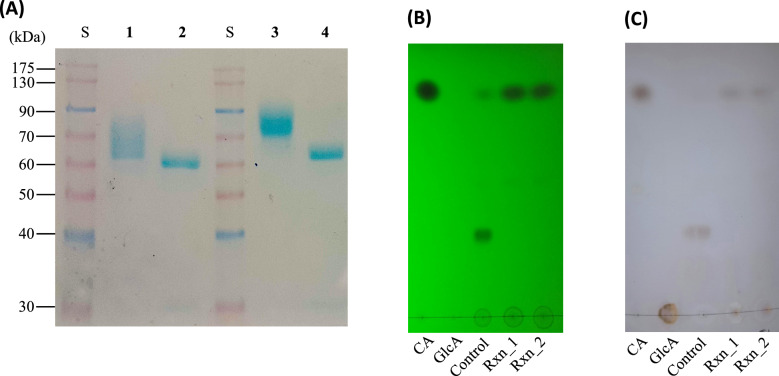
Biochemical and functional characterization of *Ae*GE15 and *Tl*GE15. SDS–PAGE of *Ae*GE15 and *Tl*GE15 (**A**) and TLC analysis of the GEs reaction mixtures using *trans*−3-phenyl-2-propen-1-yl D-glucopyranosyluronate as substrate, visualized under UV (**B**) and with N-(1-Naphthyl)-ethylenediamine dihydrochloride (**C**). *Lanes in panel* (**A**); Purified *Ae*GE15 (*1*) and *Tl*GE15 (*3*), and *Ae*GE15 (*2*) and *Tl*GE15 (*4*) after treatment with Endo H, LMW standard protein markers (*S*). *Lanes in panels* (**B**, **C**); Cinnamyl alcohol (CA), glucuronic acid (GlcA), control reaction containing the substrate without any enzyme (Control), and reactions using the purified GEs *Ae*GE15 (Rxn_1) and *Tl*GE15 (Rxn_2)

After heterologous expression, SDS–PAGE revealed MW values of 63 kDa for *Ae*GE15 and 72 kDa for *Tl*GE15 (Fig. 1A). Even after EndoH treatment, their MWs were higher than the predicted values (48.9 kDa for *Ae*GE15 and 50.0 kDa for *Tl*GE15 according to the ExPASY ProtParam tool [17]), suggesting the presence of *O*-glycosylation, most probably in the linker region rich in serine and threonine residues. Even though the *pI* of *Ae*GE15 and *Tl*GE15 are predicted to be approximately 5.3 and 5.5, respectively, the IEF–PAGE of the purified recombinant proteins revealed multiple isoforms in the 4.2–5.3 pH range for *Ae*GE15 and 4.6–5.8 for *Tl*GE15 (Figure S1), most probably due to heterogenous glycosylation, as it has been observed previously for another *O*-glycosylated modular protein from *Thermothelomyces thermophila* [22].

The GEs activity was examined on cinnamyl alcohol ester of D-glucuronic acid, which corresponds to the native bond cleaved by GEs in the plant cell wall (Fig. 1B, C). Optimal activity for *Ae*GE15 was observed at 50 °C and pH 5.0, with a strong decrease to 26% at 60 °C (Figures S2A, S2C). *Tl*GE15 acted optimally at 40 °C and pH 5.0, with 50% residual activity at 60 °C and complete activity loss at higher temperatures (Figures S2B, S2D). Of note, pH values over 6 could not be examined as the substrate is subjected to auto-hydrolysis at alkaline conditions.

Michaelis–Menten kinetic constants were determined by measuring the alcohol release by High Performance Liquid Chromatography (HPLC), after activity on cinnamyl alcohol ester of D-GlcA (Table 1). Both *Ae*GE15 and *Tl*GE15 exhibited a similar *K*_M_ to *St*GE15 from *T. thermophilus* [23]. These relatively high values of *K*_M_ could suggest low affinity of the GEs for this particular substrate, perhaps due to the absence of the 4-*Ο*-methyl group from the GlcA residue [12]. *Tl*GE15 displays the highest catalytic efficiency (*k*_*cat*_/*K*_M_) on this model substrate.

**Table 1:** Comparison of kinetic parameters of characterized GEs using *trans*−3-phenyl-2-propen-1-yl D-glucopyranosyluronate as substrate

GE	Kinetic parameters			Reactions conditions	Literature
	*K*_M_ (mM)	*k*_cat_ (s^−1^)	*k*_cat_/*K*_M_ (mM^−1^ s^−1^)		
*Ae*GE15	3.6 ± 0.6	5.4 ± 0.4	1.5 ± 0.3	50 °C/pH 5	This study
*Tl*GE15	3.2 ± 0.5	12.7 ± 0.9	4.0 ± 0.7	40 °C/pH 5	This study
*St*GE15	3.6 ± 0.6	1.9 ± 0.1	0.5 ± 0.1	50 °C/pH 6	[23]
*Pa*GE15	2.7 ± 0.5	5.3 ± 0.7	2.0 ± 0.5	50 °C/pH 6	[23]

### Structural models of GEs

**Fig. 2:**
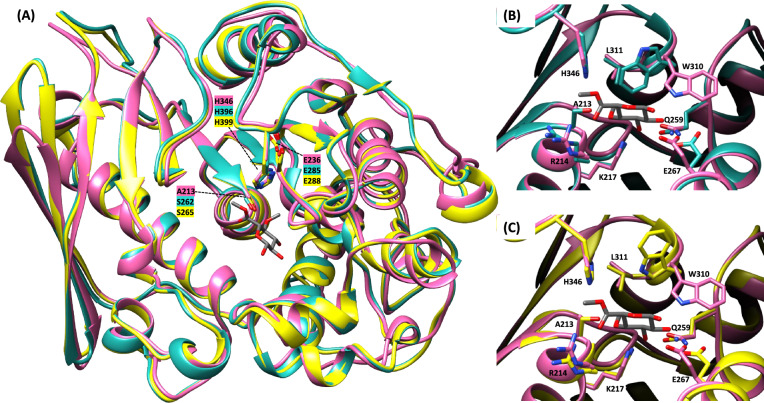
Visualization of the structural models of *Ae*GE15 (cyan) and *Tl*GE15 (yellow), comparing to the crystal structure of *St*GE15–S213A mutant (pink) in complex with methyl 4-*O*-methyl-β-D-glucopyranuronate (representation in grey sticks; PDB: 4G4J). **A** Structural models of the catalytic domain of *Ae*GE15 and *Tl*GE15 demonstrate high similarity to *St*GE15–S213A. The models of *Ae*GE15 and *Tl*GE15 were generated using AlphaFold [21]. Catalytic amino acids are marked and represented in sticks. The active sites of *Ae*GE15 (**B**) and *Tl*GE15 (**C**) reserve the amino acids that interact with the ligand (annotated and represented in sticks) in the *St*GE15–S213A structure

CE15 enzymes are α/β serine hydrolases, containing a Ser-His-Glu catalytic triad within conserved sequence motifs [10, 15, 30, 47] that were also identified in *Ae*GE15 and *Tl*GE15 (Figure S3; “V-T-G-C-**S**-R-X-G-K-G-A”, “**H**-C”, and “P-Q-**E**-S-G”; catalytic amino acids in bold), [20, 60]. GEs are divided into subgroups CE15-A and CE15-B, with the catalytic acid being positioned after the β8-strand in the former category, similar to *Ae*GE15 and *Tl*GE15 (Figure S3), or after the β7-strand in the latter group [16]. The amino acid sequence of *Ae*GE15 is similar to *Tl*GE15 (80% identity). Compared to other structurally determined GEs of the PDB database (Table S1), both *Ae*GE15 and *Tl*GE15 they exhibited the highest sequence identity with *Cu*GE15 from *C. unicolor* (6RV8; [16]), followed by *Tr*GE15 from *Trichoderma reesei* (3PIC; [47]), *Lf*GE15 from *Lentithecium fluviatile* (8B48; [34]) and *St*GE15 from *T. thermophilus* ATCC 42464 (4G4G; [10], Fig. 2A). Regarding fungal GEs, a conserved Lysine residue in *Af*GE15 from *Aspergillus fumigatus*, was suggested to contribute to the recognition of 4-*O*-methyl group of the substrate [20], while the “SGXGG” conserved region formed a consistent cavity to accommodate the 4-*O*-methyl moiety. Both of these features are identified in *Ae*GE15 and *Tl*GE15 (Figure S3). All these structural features that contribute to the MeGlcA binding in the active site of GEs could justify the low affinity of *Ae*GE15 and *Tl*GE15 for the ester bond of D-GlcA. Moreover, Trp376 of *Tt*CE15A from *Teredinibacter turnerae*, which was suggested to assist in the proper positioning of the xylan chain, was found conserved in the sequences of *Ae*GE15, *Tl*GE15, as well as *Cu*GE15 and *Af*GE15 (Figure S3), [3].

Both fungal and bacterial GEs share a similar overall fold, consisting of a three-layer sandwich [3, 10] (Fig. 2A). The surface-exposed active site of GEs (Fig. 2B, C) enables access to bulky LCC structures [12, 47]. The disulfide bonds identified in *Ae*GE15 and *Tl*GE15 (Figure S3) could result in decreased flexibility of their catalytic clefts, in contrast to the bacterial counterparts that can target more promiscuous structures [2, 3]. Both GEs were predicted to contain a CBM1 module at their N-terminal, similar to other fungal GEs [1]. CBM1s usually display a cellulose-binding function [5]. Nevertheless, affinity assays of *Ae*GE15 and *Tl*GE15 did not demonstrate binding on the examined substrates. The absence of glycosylation sites on the CBM1 domains of the studied GEs could alleviate non-specific binding, since glycosylation motifs on CBMs are suggested to strengthen interactions with cellulose [19]. Moreover, type A CBMs, including CBM1 family, interact with hydrophobic surfaces of the substrate due to the presence of aromatic amino acids within the binding sites [65]. The same hydrophobic residues of the CBM1 domain can lead to binding on aromatic moieties of lignin [59].

### Activity of GEs on pretreated beechwood biomass

Lignocellulose samples from beechwood, treated under various conditions, were tested as substrates for *Ae*GE15 and *Tl*GE15. Notably, GEs generated a product profile only from the substrate with high contents of both lignin and hemicellulose (pretreatment with distilled H_2_O/acetone (25/75%), Air, 20 bar, 160 °C for 2 h; lignin 18.7%, cellulose 50.2%, hemicellulose 19.7%; No. 4, Table S2), implying that different pretreatment methods can cause distinct structural alteration on lignocellulose, either impeding or enabling enzymatic activity. Similar observations have also been made by studying the activity of three GEs from *Sordaria brevicollis* on pretreated corn bran samples [64]. However, the effect of each pretreatment method on the LCCs linkages and the overall lignocellulosic structure of each sample remains unclear. Subsequently, pretreatment seems to play a pivotal role that requires further investigation, to optimize biomass exploitation or feature particular enzymatic activities. The substrate selected for further investigation was of high lignin (18.7%) and hemicellulose (19.7%) contents, suggesting more lignin–MeGlcA interactions that could serve as substrate for GEs due to the relatively milder pretreatment conditions compared to the other samples.

**Fig. 3:**
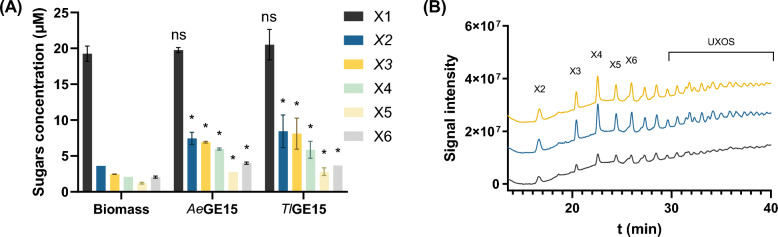
**A** XOS release by GEs activity on *An*Xyn11-treated beechwood biomass. Asterisks (*p < 0.05) indicate a significant difference based on Student’s *t* test, while “ns” labeling indicates a non-significant statistical difference. **B** HPAEC chromatogram of GEs product profile against *An*Xyn11-treated beechwood biomass (substrate: black line; *Ae*GE15: blue line; *Tl*GE15: yellow line). UXOS peaks elute after 30 min retention time. Black line: blank reaction of the solid substrate after *An*Xyn11 hydrolysis, blue line: *Ae*GE15 hydrolysate of the *An*Xyn11-treated biomass, yellow line: *Tl*GE15 hydrolysate of the *An*Xyn11-treated biomass

After exhaustive GH11 xylanase treatment of substrate No. 4, the easily accessible xylan was considered to be removed. GH11 xylanases are low molecular weight enzymes that penetrate complex lignocellulosic substrates and attack β−1,4-glycosidic linkages between Xyl*p* residues of less substituted regions of xylan [28, 41]. Therefore, the biomass fraction contains the recalcitrant part of glucuronoxylan that remains attached to lignin via LCCs, while the xylanase hydrolysate contains various soluble aldouronic acids, including longer fractions, possibly linked to aromatic moieties. Activity of the GEs was examined on both of these fractions. Neither of them altered the product profile in the liquefied fraction, in accordance with previous observations that these enzymes act on insoluble substrates [37, 57]. However, upon activity on the solid fraction, GEs increased the release of neutral xylooligosaccharides (XOS) and uronic xylooligosaccharides (UXOS) (Fig. 3A, B), although at low concentration. Even though the release of UXOS is expected, as indicated by the proposed activity of GEs, the release of XOS cannot be justified based on current knowledge, even though it has been observed before [38]. This would imply the existence of ester bonds between hemicellulose sugars and phenolic acids, which has not been described to date. At least 6.8 ± 0.1% of hemicellulose is hydrolyzed by *An*Xyn11, based on xylose equivalents of xylose to xylotriose (X1–X3) measured in the hydrolysate fraction by high-performance anion exchange chromatography (HPAEC). During hydrolysis of the biomass fraction, GEs increased saccharification up to 57 ± 1 μM (*Ae*GE15) and 61 ± 3 μM (*Tl*GE15) of xylose equivalents of X1–X6 sugars. Most importantly, a wide variety of unidentified UXOS and longer sugars are also released by the GEs, which both exhibit a similar product profile (Fig. 3B). These oligomers could be embedded in the plant cell wall matrix and are released when the CE15s cleave the LCC bonds.

The catalytic mechanism of GEs involves a two-stage reaction [62], during which a non-covalent acyl-serine intermediate is formed, following the deacylation and the serine regeneration. Even though recent QM/MM studies on *Ot*CE15A indicated the deacylation of the enzyme-acyl intermediate as the rate-limiting step [66], the rate of enzymatic dissociation from bulkier substrate complexes, based on in silico analysis, appears to be slower. The dissociation process of the GE–carbohydrate complex could be affected by the physicochemical properties of the complexed carbohydrate fraction. Therefore, the rate-limiting step of the GE catalytic cycle may differ depending on the exact LCC structure being attacked. Both *Ae*GE15 and *Tl*GE15 were suggested to act on bulky structures of the solid *An*Xyn11-treated residue, and thus the dissociation process could be the rate-limiting step. Thus, reactions were incubated for 24 h for the experiments.

### Synergistic relationships with a GH11 xylanase on pretreated beechwood

**Fig. 4:**
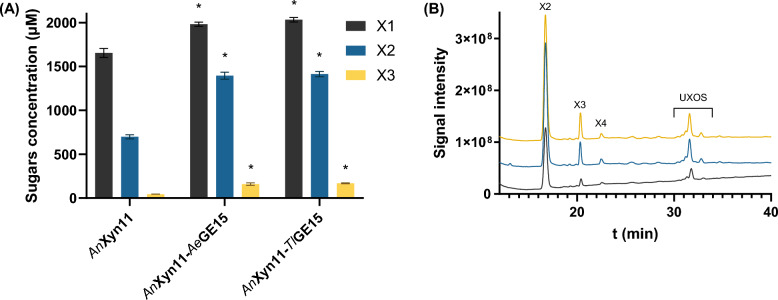
**A** Effect of *Ae*GE15 and *Tl*GE15 on the concentration of sugars released by *An*Xyn11 during hydrolysis of pretreated beechwood. **B** Effect of *Ae*GE15 and *Tl*GE15 on the product profile of *An*Xyn11 during cooperative hydrolysis of pretreated beechwood (*An*Xyn11: black line; *An*Xyn11–*Ae*GE15: blue line; *An*Xyn11–*Tl*GE15: yellow line)

*Ae*GE15 and *Tl*GE15 demonstrated different effects on distinct xylanase specificities. Regarding activity on pretreated beechwood, no cooperation was observed with *Tm*Xyn10, which shows an increased tolerance of substituted residues in the catalytic cleft [35, 43]. On the contrary, the cooperation with *An*Xyn11 led to an enhanced release of XOS and UXOS. A 20 ± 3% increase in X1 by *Ae*GE15 and 23 ± 3% by *Tl*GE15 was observed, for xylobiose (X2) the corresponding values were 100 ± 7% and 102 ± 6%, while X3 release was significantly promoted by the GEs, reaching a 254 ± 40% increase by *Ae*GE15 and 272 ± 15% by *Tl*GE15 (Fig. 4A, B). Nevertheless, X3 concentrations were evidently lower, reaching a maximum of 160 μM, compared to X1 and X2 concentrations, which reached a maximum of 2 mM (Fig. 4A). A prominent increase in the intensity of peaks was also observed for products corresponding to aldouronic acids (Fig. 4B). Interestingly, hydrolysis of hemicellulose by *An*Xyn11, based on xylose equivalents of unsubstituted X1–X3, was increased from 6.1 ± 0.1% to 10.1 ± 0.2% and 10.3 ± 0.1% in the presence of *Ae*GE15 and *Tl*GE15, respectively. Even though GH11 xylanases tolerate MeGlcA substitutions within subsites − 3 and + 2 of the catalytic cleft, their activity is unknown when this particular side-group is involved in LCC linkages [35, 61]. The esterified MeGlcA-substituted Xyl*p* residue could inhibit xylanases, while GEs could disrupt the ester linkages and enable GH11 activity, according to their established mode of action.

Notably, the product release during enzymatic hydrolysis of the pretreated beechwood is low, which could be explained by a number of factors. The non-selective binding of the biocatalysts to lignin could partly explain this limited enzymatic activity. Evidently, the concentration of *Tt*CE15A from *T*. *turnerae* was decreased by 30% due to adsorption on lignin, while a 70% reduction was observed for α-glucuronidases during incubation on insoluble LCC fractions [48]. Non-specific binding of enzymes to aromatic groups of lignin could be counteracted by non-ionic detergents, to improve the solubility of complex lignocellulosic substrates and accessibility by GEs or even using protein engineering to modify hydrophobic surfaces of the enzyme that preferably adsorb onto lignin. Moreover, inhibition of GEs by soluble aromatic compounds, such as hydroxycinnamic acids, has also been reported [3]. Another concern is that measuring the sugar release alone cannot fully demonstrate the synergy between GEs and xylanases, which could lead to the release of structurally complex oligomers that are difficult to identify. Moreover, other enzymatic specificities may also be required to illustrate GEs activity properly, due to their limited accessibility to the LCC esters.

### Synergy of GEs with a GH30 glucuronoxylanase/xylobiohydrolase on pretreated beechwood

**Fig. 5:**
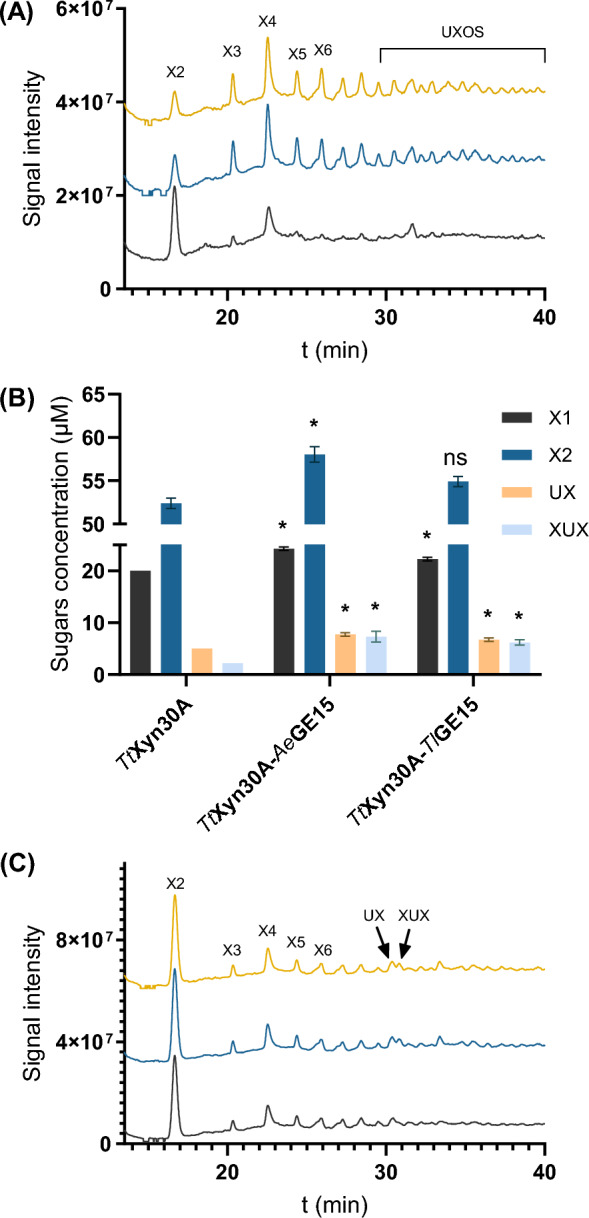
**A** HPAEC chromatogram of GEs product profile against *An*Xyn11–*O*CE6-treated beechwood (substrate: black line; *Ae*GE15: blue line; *Tl*GE15: yellow line). **B** Sugar release during synergistic relationships of GEs with *Tt*Xyn30A on *An*Xyn11–*O*CE6-treated beechwood. Asterisks (*p < 0.05) indicate a significant difference based on Student’s *t* test, while “ns” labeling indicates a non-significant statistical difference. **C** Cooperative activity of *Tt*Xyn30A with GEs on *An*Xyn11–*O*CE6-treated beechwood (*Tt*Xyn30A: black line; *Tt*Xyn30A–*Ae*GE15: blue line; *Tt*Xyn30A–*Tl*GE15: yellow line)

The effect of GEs on biomass hydrolysis by the bi-functional *Tt*Xyn30A was examined. *Tt*Xyn30A is a glucuronoxylanase that binds to the MeGlcA-substituted Xyl*p* residue in xylan and hydrolyzes the glycosidic bond of the adjacent Xyl*p* towards the reducing end, while it also releases xylobiose from the non-reducing end, leading to the final hydrolysis products of X2, 2^2^-(4-O-methyl-α-D-glucuronosyl-)-xylobiose (UX) and 2^2^-(4-O-methyl-α-D-glucuronosyl-)-xylotriose (XUX) [24]. In this case, a xylanase–acetyl xylan esterase combination was used to remove synergistically part of the carbohydrate fraction from the pretreated beechwood substrate, revealing more esterified MeGlcA units that could serve as substrate for *Tt*Xyn30A. The pretreated substrate (No. 4) contained approximately 5% (w/w) acetic acid. The deacetylation of the remaining carbohydrate part in the surroundings of the MeGlcA-linkages could also facilitate *Tt*Xyn30A activity [44]. The *An*Xyn11–*O*CE6 treatment led to an approximately 7% hydrolysis of hemicellulose. Interestingly, activity of GEs on the *An*Xyn11–*O*CE6-treated biomass resulted in diverse peaks corresponding to UXOS (Fig. 5A), along with an increased XOS release. Regarding *Tt*Xyn30A activity, synergistic effects were displayed only on the enzymatically treated biomass fraction. A slight increase was observed in X1 (21 ± 2% and 11 ± 2%) and X2 (11 ± 2% and 5 ± 1%) release, in the presence of *Ae*GE15 and *Tl*GE15, respectively, whereas a more prominent increase was noted for UX by 55 ± 8% and 35 ± 7%, respectively, and XUX by 233 ± 57% and 183 ± 28% (Fig. 5B, C). In detail, X1 was increased from 20 ± 0 μM to 24.3 ± 0.4 (*Ae*GE15) and 22.3 ± 0.4 μM (*Tl*GE15), X2 from 52.4 ± 0.6 μM to 58.1 ± 0.9 (*Ae*GE15) and 54.9 ± 0.6 μM (*Tl*GE15), whereas in the presence of GEs the UX release was raised from 5 ± 0 μM to 7.8 ± 0.4 (*Ae*GE15) and 6.8 ± 0.4 μM (*Tl*GE15) and XUX was increased from 2.2 ± 0.0 μM to 7.3 ± 1.0 (*Ae*GE15) and 6.2 ± 0.5 μM (*Tl*GE15). These results indicate that GEs reveal additional acting-sites mainly for the endo-activity of *Tt*Xyn30A, while the lesser effect on xylobiohydrolase activity could be attributed to the removal of the linear part of xylan by the formerly acting *An*Xyn11–*O*CE6 system.

Even though GEs have been shown to promote biomass saccharification by facilitating substrate access to xylanases, most of the experiments have been conducted on lignin-rich derivatives from birchwood, with *Cu*GE being the most extensively studied CE15 representative. Activity of *Cu*GE (0.15 mg mL^−1^) on LCCs from birchwood [lignin-rich precipitate (LRP), 5 mg mL^−1^] released longer acetylated UXOS, which were further hydrolyzed by a commercial GH10 endo-xylanase (0.05 mg mL^−1^), resulting in higher product release compared to that achieved by the xylanase alone [38]. Approximately 30 μM MeGlcA equivalents and 120 μM xylose equivalents were released by the esterase–xylanase combination, in contrast to 10 and 80 μM, respectively, released only by the xylanase. Moreover, *Cu*GE releases heavily substituted acetylated UXOS of a high degree of polymerization (average degree of polymerization 30) from insoluble LRP from birch, which further serves as a substrate for a commercial GH10 endo-xylanase [37]. To our knowledge, there have been no studies against beechwood samples. As demonstrated in this work, both *Ae*GE15 and *Tl*GE15 act on the recalcitrant part of beechwood glucuronoxylan, which remains attached to lignin, even after xylanase hydrolysis, while synergistic degradation was observed both with *An*Xyn11 and *Tt*Xyn30A.

### Cooperative degradation of pretreated corn bran by GEs and a GH10 xylanase

**Fig. 6:**
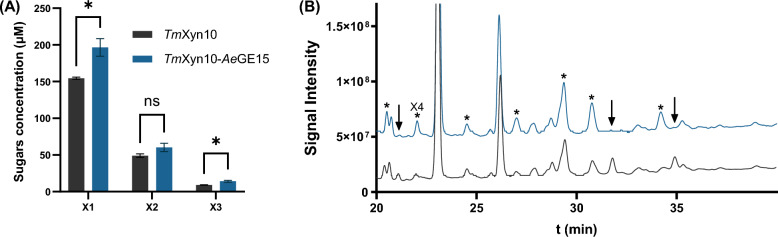
Cooperative activity of *Tm*Xyn10 and *Ae*GE15 on DSCB. **A** Effect of *Ae*GE15 on sugars release by *Tm*Xyn10. Asterisks (*p < 0.05) indicate a significant difference based on Student’s *t* test, while “ns” labeling indicates a non-significant statistical difference. **B** HPAEC chromatogram demonstrating the effect of *Ae*GE15 on the product profile of *Tm*Xyn10 (*Tm*Xyn10: black line; *Tm*Xyn10–*Ae*GE15: blue line). Increased peaks are indicated by asterisks, while the decreased peaks by downward arrows

Investigation of the cooperative hydrolysis of pretreated destarched corn bran (DSCB) by xylanases and GEs demonstrated a synergistic effect only for the *Tm*Xyn10–*Ae*GE15 combination. Glucuronoarabinoxylan, the main hemicellulose of DSCB, is a complex, highly substituted polymer with increased heterogeneity regarding the side-chain decorations, recalcitrant to xylanase hydrolysis [39, 49]. Despite the rather relaxed specificity of GH10 xylanases, it is unclear whether the esterified MeGlcA substitutions could be accommodated in the − 2 or + 1 subsites, impeding xylanolytic activity. Supplementation by *Ae*GE15 resulted in a 27 ± 8% increase in X1 release and a 55 ± 15% increase for X3 (Fig. 6Α, Β), while a minor release of xylotetraose (X4; 4 μΜ) was also observed.

Several studies have been focused on the cooperative degradation of pretreated corn samples, investigating the effect of GEs on enzymatic cocktails. A boosting effect by *Cu*GE and *Tr*GE (from *T. reesei*) (25 μg mL^−1^ each) on two commercially available hemicellulase and cellulase preparations (125 μg mL^−1^ each) in combination with a GH3 β-xylosidase from *T*. *reesei* (25 μg mL^−1^) have been demonstrated during hydrolysis of pretreated corn fiber (25 mg mL^−1^) [11]. These relatively high enzyme concentrations are essential, considering the intimate cellulose–hemicellulose–lignin interactions that impeded accessibility of GEs to LCC linkages. Both esterases resulted in a 5–10% increase of the total sugar release (xylose, arabinose, glucose and GlcA) by each enzymatic preparation. Similarly, supplementation of a commercial cellulolytic–hemicellulolytic cocktail with GEs (*Sl*CE15A, *Su*CE15A or *Su*CE15C) upon hydrolysis of ball-milled corn cob (5 mg mL^−1^) led to increased release of glucose (90–300%), xylose and arabinose (20–50%) [2], achieving a maximum of approximately 20 μg mL^−1^ arabinose, 60 μg mL^−1^ xylose and 125 μg mL^−1^ glucose by *Su*CE15C addition. Another synergy was observed on the autohydrolysis residues of corn bran (25 mg mL^−1^), between *Tt*GE1 (0.1 mg mL^−1^) or *Tt*GE2 (0.15 mg mL^−1^) from *Thielavia terrestris* and an endo-1,4-β-xylanase preparation from *Trichoderma longibrachiatum* (0.3 mg mL^−1^) [57], resulting in increased release of glucuronic acid (9.2% and 4.0%, respectively), arabinose (92.6% and 51.9%), galactose (58.8% and 54.8%), glucose (43.5% and 36.3%) and xylose (39.9% and 42.1%).

Nevertheless, the effect of GEs on the catalytic activities of individual hemicellulose-targeting enzymes rather than enzymatic cocktails has been less extensively studied. The synergy between *Tt*GE15A from *T*. *turnerae* and two α-glucuronidases has been demonstrated on LCCs from birchwood (5 mg mL^−1^), after prolonged incubation (72 h) [48]. The combined activity of the GE with *Sde*Agu115A (*Saccharophagus degradans*) led to an increase in MeGlcA release from 52 to 67%, while the cooperative activity with *Axy*Agu115A (*Amphibacillus xylanus*) resulted in a corresponding increase from 61 to 95%. This work, however, demonstrates the effect of *Ae*GE15 and *Tl*GE15 on the hydrolysis of mildly pretreated corn bran by three distinct xylanases. In contrast to pretreated beechwood, the only beneficial effect was observed for *Tm*Xyn10, implying that distinct synergistic effects may be observed for the same enzymatic system, acting on biomasses of different origin. Similar synergistic effects between a GH10 xylanase and three GEs in the presence of an α-glucuronidase has also been reported during hydrolysis of pretreatred corn bran [64]. In addition, the inability of *Tl*GE15 to promote *Tm*Xyn10 hydrolysis, as opposed to *Ae*GE15, could imply distinct tolerance to substitutions between the two esterases, which becomes apparent on native lignocellulosic structures that incorporate rather elaborate interactions between the various polymeric chains [26, 39]. Besides, multiple CE15 genes in microorganisms are suggested to exhibit distinct specificities and biological functions, as they have also been found to display different transcriptional responses to different growth conditions [2].

## Conclusions

The MeGlcA–lignin associations significantly contribute to the overall recalcitrance of biomass against enzymatic hydrolysis. The diverse linkages between carbohydrates and aromatic moieties add to the overall heterogeneity of LCCs, while the complex interactions of lignin with cellulose and hemicellulose are not yet fully understood, hampering the efficient exploitation of biomass-degrading enzymes. Since emphasis has been given to the extraction and industrial exploitation of lignin, GEs are considered a valuable asset for polishing this aromatic polymer, as a “green” alternative to chemical treatment. Their exact mode of action, however, on native structures has not been determined, limiting their applicability in industrial bioprocesses. In this work, the activity of two fungal GEs from the CE15 family was investigated on pretreated beechwood. We demonstrated that these fungal GEs display different synergistic relationships with distinct xylanase families (GH10, GH11, GH30), depending on the lignocellulosic material. This suggests that the structural and compositional features of the targeted biomass must be taken into account to select the appropriate enzymes for biomass biodegradation. Finally, a novel synergistic relationship was demonstrated between the GEs and the bifunctional *Tt*Xyn30A, paving the way for a more efficient production of substituted XOS from lignocellulosic biomass. A more thorough study on the cooperative relationships between GEs and different cell wall-degrading enzymes is needed to gain a better understanding of their potential in the design of effective enzymatic cocktails for future lignocellulose biorefineries.

## Materials and methods

### Enzymes and chemicals

The GH10 endo-1,4-β-xylanase *Tm*Xyn10 from *Thermotoga maritima*, GH11 endo-1,4-β-xylanase *An*Xyn11 from *Aspergillus niger*, CE6 acetyl xylan esterase *O*CE6 from *Orpinomyces* sp., glucuronoyl esterase assay kit (K-GEUX3), glucuronoxylan (beechwood), arabinoxylan (rye flour) and insoluble wheat arabinoxylan were purchased from Megazyme (Bray, Co. Wicklow, Ireland). Avicel and carboxymethyl cellulose of low viscosity were acquired from Sigma-Aldrich (St. Louis, MO, USA). Other chemicals, supplied from Applichem (Darmstadt, Germany) or Sigma-Aldrich, were of the highest purity available. The pretreated beechwood was derived from commercially available Lignocel^®^ HBS 150–500 by processing under various conditions (Table S2), [25]. Corn bran material was gifted from Karanikas Mills S.A. (Alexandria Imathias, Greece). The particle size of the granules was reduced to less than 5 mm in diameter using a benchtop grinder-mill. De-starching of biomass involved incubation of 300 mL of 10% (w/v) corn with 1 mL α-amylase-containing Liquozyme SCDS (Novozymes, Denmark) preparation in 50 mM potassium–acetate buffer, pH 6.0, at 80 °C for 2 h. The pH was then adjusted to 5.0 with acetic acid and the mixture was supplemented with amyloglucosidase-containing Spirizyme Fuel (Novozymes; 0.1% w/w biomass), following incubation at 60 °C for 1 h. The solid residue was washed four times with equal volumes of distilled water (60 °C) and dried at 60 °C for 16 h. The de-starched substrate [10% (w/v)] was further pretreated with 3% (v/v) acetic acid at an autoclave under 20 bars at 100 °C for 1 h. The pretreated biomass was further washed 3 times with distilled water and was dried at 60 °C for 16 h. Compositional analysis, following the NREL protocol [52] and the released sugars were quantified by HPLC, using a set of Cation-H Cartridge and Anion-CO_3_ Micro Guard columns and a Micro-Guard Carbo-P column (30 mm × 4.6 mm, Bio-Rad; Hercules, CA, USA) connected to an Aminex HPX-87P column (300 mm × 7.8 mm, Bio-Rad) at 85 °C, and an RID 10A detector, using ultrapure H_2_O at a flow rate of 0.6 mL·min^−1^, retrieving samples of 10 μL. Acetic acid content of the substrates was estimated by alkali treatment of lignocellulose (10 mg) with NaOH (4 mL, 4 M) for 16 h at room temperature in the dark, as described previously [45]. The final pretreated DSCB material consisted of 22% (w/w) glucose, 20% xylose, 9% arabinose, 3.5% galactose, 6.5% acid-insoluble lignin and 13.5% acetic acid. The synthesis of cinnamyl alcohol ester with D-glucuronic acid was performed enzymatically by immobilized lipase B (Novozym 435) from *C. antarctica* [23], and was verified by Thin Layer Chromatography (TLC), using aluminum-coated silica gel 60 F254 plates (Merck, Germany), and a mixture of chloroform:methanol:water 65:15:20 (v/v/v) as resolving solution. Chemical compounds were visualized either with UV absorbance at 254 nm or by applying 6.5 mM N-(1-Naphthyl)-ethylenediamine dihydrochloride in methanol and 3% (v/v) sulfuric acid [9] followed by heating at 100 °C for 10 min. Alcohol release was quantified by HPLC (Jasco PU 987) on a reverse phase C-18 Nucleosil column (250 mm × 4.6 mm, Macherey–Nagel, Germany), performing elution with a mixture of methanol/water (7:3, v/v) at a flow rate of 0.4 mL min^−1^ using a UV detector Jasco UV 975 at 254 nm. Bifunctional MeGlcA-dependent xylanase/xylobiohydrolase *Tt*Xyn30A from *T. thermophila* was produced and purified according to Katsimpouras et al., [24].

### GEs heterologous expression and biochemical characterization

Two GE genes were selected from the basidiomycetes *A. elegans* and *T. ljubarskyi*, namely, *aege* (1639 bp; Protein ID 369508) and *tlge* (1643 bp; Protein ID 962604). The signal peptide was predicted using the SignalP 4.0 server [58], while potential *N*- and *O*-glycosylation sites were predicted using the Net*N*Glyc 1.0 and Net*O*Glyc 4.0 servers [8, 55]. The proteins were produced using the in-house 3PE platform (*Pichia pastoris* protein express; www.platform3pe.com). The nucleotide sequences coding for *Ae*GE15 and *Tl*GE15 were synthesized after codon optimization for expression in *P. pastoris* (syn. *Komagataella phaffii*). Each gene was inserted into the expression vector pPICZαA (Invitrogen®, Carlsbad, California, USA) using *XhoI* and *XbaI* restriction sites in frame with the α-secretion factor at the N-terminus (i.e., without native signal peptide) and with a (His)_6_-tag at the C-terminus (without *c*-myc epitope) (Genewiz^®^, Leipzig, Germany). Transformation of competent *P. pastoris* X33 and selection of zeocin-resistant *P. pastoris* transformants screened for protein production were carried out as previously described [18]. The best-producing transformants were conserved as glycerol stock at − 80 °C. The GEs were heterologously expressed as described by Katsimpouras et al., [24], in BMMY medium by 0.5% (v/v) methanol induction within 5 day incubation at 30 °C and 180 rpm. The cells were removed by centrifugation and the supernatant was collected and filtrated with 0.2 μm filters (Supor^®^ 200, PALL Life Sciences). Protein purification was achieved through Immobilized Metal Affinity Chromatography and the enzyme preparation was then dialyzed in 20 mM Tris–HCl pH 8.0. Potential *Ν*-glycosylation sites of the recombinant proteins were investigated by treatment with endo-glucosidase Η (EndoH, New England Biolabs, Ipswich, MA, USA), according to the commercial protocol. Enzyme expression and purification were validated by sodium dodecyl sulphate–polyacrylamide gel electrophoresis (SDS–PAGE). The isoelectric point (p*I*) was determined by isoelectric focusing–polyacrylamide gel electrophoresis (IEF–PAGE) using a PhastSystem electrophoresis unit (Amersham Biosciences Corp., Sweden).

The optimal temperature for each GE was examined at a temperature range of 10 to 80 °C for 15 min using cinnamyl alcohol ester of D-glucuronic acid. The optimal pH was investigated at a range of 3.0 to 11.0 at optimum temperature for 15 min. For this reason, the following buffering systems were used: citrate–phosphate (pH 3.0–7.0), Tris–HCl (pH 8.0–9.0) and glycine–NaOH (pH 10.0–11.0). The thermal stability of the proteins was examined by measuring the residual enzymatic activity after incubation at 30 to 70 °C in 100 mM phosphate–citrate pH 5 for 24 h. Stability at different pH was measured after incubating the protein in the different buffering systems at 4 °C for 24 h. The Michaelis–Menten model was used to study the kinetics of the GEs activity. The esterases were studied on cinnamyl alcohol ester of D-glucuronic acid at 0.2–5.0 mM under optimal reaction conditions. The Michaelis–Menten kinetic constants were estimated using the GraphPad Prism v. 6, GraphPad Software (La Jolla, CA, U.S.A.). The effect of various metal ions and chemicals (Ca^2+^, Co^2+^, Ni^2+^, Zn^2+^, Na^+^, Cu^2+^, Ag^2+^, Mg^2+^, Mn^2+^, Fe^3+^, EDTA, urea, Triton X-100, acetone and SDS) on each GE activity was evaluated after incubating the enzymes with these compounds (1 mM, 5 mM and 10 mM) at 25 °C for 2 h and measuring afterward their residual activity under optimal conditions in the presence of each compound.

### Structural models of GEs

The 3D structural models of *Ae*GE15 and *Tl*GE15 were constructed using AlphaFold [21]. UCSF Chimera (V:1.17.1) [46] was used for structure visualization and figure preparations. The carbohydrate-binding module (CBM) prediction was achieved with CBMDB online tool [32]. Sequence alignment was performed for both esterases within the Protein Data Bank (PDB) database [6], using the online NCBI Blast tool. The multiple structure-based sequence alignment of *Ae*GE15, *Tl*GE15, *Af*GE15 (EAL89275.1) and *Cu*GE15 (PDB code: 6RV8) was prepared using the Clustal Omega multiple sequence alignment tool [33], and visualized with ESPript 3.0 [50].

### CBM affinity assays

The pull-down assay involved incubation of each esterase (0.35 mg mL^−1^) with the insoluble polymeric substrate (Avicel and insoluble wheat arabinoxylan, at 2% (w/v) concentration) in pH 5 acetate buffer (sodium acetate/acetic acid) 50 mM for 4 h at 4 °C under mild agitation. Supernatant was collected after a 10-min centrifugation, while the solid residue was washed sequentially with water and SDS sample buffer, collecting the supernatant at each step. Protein detection in the samples was achieved with SDS–PAGE electrophoresis [29]. Affinity nondenaturing gel electrophoresis (ANDE) was performed to investigate the ability of the CBM1 module of *Ae*GE15 and *Tl*GE15 to bind on soluble polysaccharides (beechwood glucuronoxylan, rye arabinoxylan, CMC). For each polymeric substrate, a set of 2 gels was prepared using 7.5% acrylamide in 25 mM Tris and 250 mM glycine buffer (pH 8.3). One of the gels, was supplemented with 0.1% (w/v) soluble polysaccharide. Bovine Serum Albumin was used as a negative control. Protein samples (5–10 μg each) were diluted in approximately 50% (v/v) glycerol and 0.1% (w/v) bromophenol blue dye. Gels were run at 10 mA per gel for 2 h. Coomassie brilliant blue G-250 reagent was used for gel staining.

### Activity of GEs on beechwood biomass

Activity of GEs was initially screened on beechwood samples that have been previously pretreated under different conditions (Table S2). Each pretreated beechwood sample (35 mg mL^−1^) was treated with *Ae*GE15 or *Tl*GE15 (20 μg mL^−1^ each) in 50 mM acetate buffer (sodium acetate/acetic acid), pH 5, at 45 °C and 950 rpm for 24 h using an Eppendorf Thermomixer Comfort (Eppendorf, Germany). After thermal inactivation of the GEs, sugar analysis was conducted with HPAEC, as described by Pentari et al., [44]. Subsequently, one of the lignocellulose samples (No 4, Table S2) was selected, to further investigate the specificity of each GE separately on the hydrolysate as well as the solid fraction of the xylanase-treated biomass. The selected pretreated beechwood (35 mg mL^−1^) was treated with *An*Xyn11 (0.2 mg mL^−1^) in 50 mM acetate buffer, pH 5, at 45 °C and 950 rpm for 72 h. After a 10 min boiling for thermal inactivation of the xylanase, reaction mixtures were centrifuged, the supernatant was collected and the remaining biomass was washed twice with distilled H_2_O and collected via centrifugation. The hydrolysate was further divided into aliquots of 400 μL, while biomass samples were resuspended in 50 mM acetate buffer, pH 5. Both of the hydrolysis fractions (solubilized and solid) that would potentially serve as GE substrates were supplemented with *Ae*GE15 or *Tl*GE15 (40 μg mL^−1^ each) and incubated at 45 °C and 950 rpm for 24 h. After thermal inactivation of the reaction mixtures, sugar analysis was performed with HPAEC.

### Synergistic relationships with different xylanases on pretreated lignocellulosic substrates

Synergistic relationships of GEs with xylanases of different GH families were further examined on pretreated beechwood. Lignocellulosic biomass (35 mg mL^−1^) was treated with binary combinations of *Tm*Xyn10 or *An*Xyn11 (0.2 mg mL^−1^) with either of the *Ae*GE15 or *Tl*GE15 (40 μg mL^−1^ each), in 50 mM acetate buffer, pH 5, at 45 °C and 950 rpm for 24 h. Due to limited activity of *Tt*Xyn30A on this particular substrate, the xylanase–GE cooperative effect was examined on the *An*Xyn11–*O*CE6-treated biomass fraction as well as the corresponding hydrolysate. The enzymatically treated biomass and hydrolysate were prepared under the same reaction conditions, using both *O*CE6 (40 μg mL^−1^) and *An*Xyn11 (0.2 mg mL^−1^) for 24 h. After thermal inactivation of the *An*Xyn11–*O*CE6 combination, the reaction of *Tt*Xy30A (0.2 mg mL^−1^) with either of the GEs (40 μg mL^−1^) took place for another 24 h under the same conditions. Analysis of XOS and UXOS was achieved with HPAEC, after thermal inactivation of the enzymes in each sample. The same experimental procedure was applied using DSCB as substrate, with the difference of supplementing the reaction mixture with GEs at increased concentration (0.1 mg mL^−1^).

### Statistical analysis

All reactions were performed in duplicates in 600 μL reaction volumes. Appropriate blank reactions, without the corresponding enzymes, were run in parallel for each condition. Results are presented as a mean value and standard deviation. Statistical analysis was performed with GraphPad Prism 10.0.2 (GraphPad Software, Inc., U.S.A.).

## Supplementary Information

Additional file 1. Exploring the synergy between fungal CE15 glucuronoyl esterases and xylanases for lignocellulose saccharification” includes Supplementary Figs. 1–3 and Supplementary Tables 1 and 2. Supplementary Fig. 1. IEF of *Ae*GE15 and *Tl*GE15. Lanes; Purified *Ae*GE15 (1) and *Tl*GE15 (3), and *Ae*GE15 (2) and *Tl*GE15 (4) after treatment with Endo H, standard protein markers with pI range 3.0–10.0 (M). Supplementary Fig. 2. Effect of temperature and pH on the activity of *Ae*GE15 (A, C) and *Tl*GE15 (B, D) on cinnamyl alcohol ester of D-glucuronic acid. All assays were carried out in duplicates. Supplementary Fig. 3. Structure-based sequence alignment of *Ae*GE15, *Tl*GE15, *Af*GE15 and *Cu*GE15. Secondary structure elements are drawn as black arrows (β-sheets) and black spirals (α-helixes). The 3_10_-helixes are labeled as “η”. Strict β-turns are rendered with TT letters. Blue frames indicate similarity across groups. Identical and similar residues are printed in white on a red background and in red on a white background, respectively. The conserved motifs that include catalytic amino acids are indicated by green lines and green triangles, respectively. The Lysine residue suggested to interact with the MeGlcA moiety and the “SGXGG” motif that forms the cavity for its accommodation are denoted by a yellow star and a yellow line, respectively. The conserved Tryptophan of *Tt*GE15A is rendered with a red star. Green digits resemble disulfide bridges and grey stars indicate residues with alternate conformations. Supplementary Table 1. Identity values among the amino acid sequences of *Ae*GE15 and *Tl*GE15, compared to other mature GEs from different microorganisms. Supplementary Table 2. Different pretreatment methods of beechwood biomass and the compositional analysis of the derived samples. Data have been obtained from [25].

## Data Availability

No datasets were generated or analysed during the current study.
