# Supplementary material for: Exploring the synergy between fungal CE15 glucuronoyl esterases and xylanases for lignocellulose saccharification
Source: Biotechnol Biofuels Bioprod. 2025 Mar 26;18:38. doi: 10.1186/s13068-025-02639-0 (PMC11948903; doi:10.1186/s13068-025-02639-0)
Supplement: Supplementary file 1 — Additional file 1. Exploring the synergy between fungal CE15 glucuronoyl esterases and xylanases for lignocellulose saccharification” includes Supplementary Figs. 1–3 and Supplementary Tables 1 and 2. Supplementary Fig. 1. IEF of AeGE15 and TlGE15. Lanes; Purified AeGE15 (1) and TlGE15 (3), and AeGE15 (2) and TlGE15 (4) after treatment with Endo H, standard protein markers with pI range 3.0–10.0 (M). Supplementary Fig. 2. Effect of temperature and pH on the activity of AeGE15 (A, C) and TlGE15 (B, D) on cinnamyl alcohol ester of D-glucuronic acid. All assays were carried out in duplicates. Supplementary Fig. 3. Structure-based sequence alignment of AeGE15, TlGE15, AfGE15 and CuGE15. Secondary structure elements are drawn as black arrows (β-sheets) and black spirals (α-helixes). The 310-helixes are labeled as “η”. Strict β-turns are rendered with TT letters. Blue frames indicate similarity across groups. Identical and similar residues are printed in white on a red background and in red on a white background, respectively. The conserved motifs that include catalytic amino acids are indicated by green lines and green triangles, respectively. The Lysine residue suggested to interact with the MeGlcA moiety and the “SGXGG” motif that forms the cavity for its accommodation are denoted by a yellow star and a yellow line, respectively. The conserved Tryptophan of TtGE15A is rendered with a red star. Green digits resemble disulfide bridges and grey stars indicate residues with alternate conformations. Supplementary Table 1. Identity values among the amino acid sequences of AeGE15 and TlGE15, compared to other mature GEs from different microorganisms. Supplementary Table 2. Different pretreatment methods of beechwood biomass and the compositional analysis of the derived samples. Data have been obtained from [25]. [file 13068_2025_2639_MOESM1_ESM.docx]

Additional file 1

**Exploring the synergy between fungal CE15 glucuronoyl esterases and xylanases for lignocellulose saccharification**

Christina Pentari^1^, Constantinos Katsimpouras^2^, Mireille Haon^3^, Jean-Guy Berrin^3^, Anastasia Zerva^4^, Evangelos Topakas^1*^

^1^*Industrial Biotechnology & Biocatalysis Group, School of Chemical Engineering, National Technical University of Athens, 9 Iroon Polytechniou Str., Zografou Campus, Athens 15772, Greece*

^2^ Department of Chemical Engineering, Massachusetts Institute of Technology, 77 Massachusetts Avenue, Cambridge, 02139 MA, USA

^3^ *INRAE, Aix Marseille Univ., BBF, Biodiversité et Biotechnologie Fongiques, 13009 Marseille, France*

^4^ *Laboratory of Enzyme Technology, Department of Biotechnology, School of Applied Biology and Biotechnology, Agricultural University of Athens, 75 Iera Odos Street, 11855 Athens, Greece*

* Correspondence: Evangelos Topakas, [vtopakas@chemeng.ntua.gr](mailto:vtopakas@chemeng.ntua.gr)


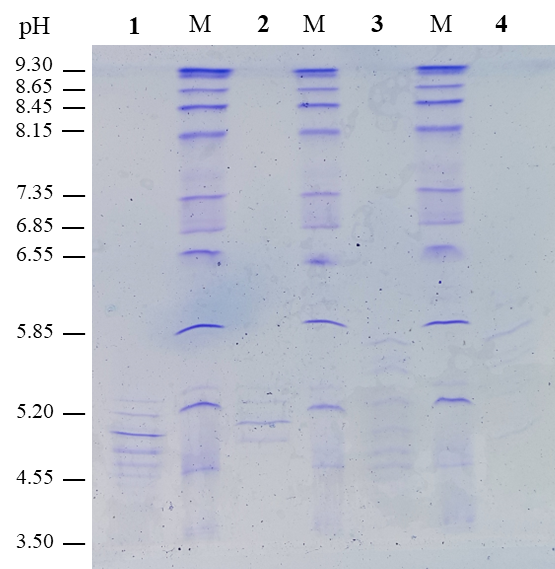


Supplementary Figure 1. IEF of *Ae*GE15 and *Tl*GE15. *Lanes*; Purified *Ae*GE15 (*1*) and *Tl*GE15 (*3*), and *Ae*GE15 (*2*) and *Tl*GE15 (*4*) after treatment with Endo H, standard protein markers with p*I* range 3.0-10.0 (*M*).


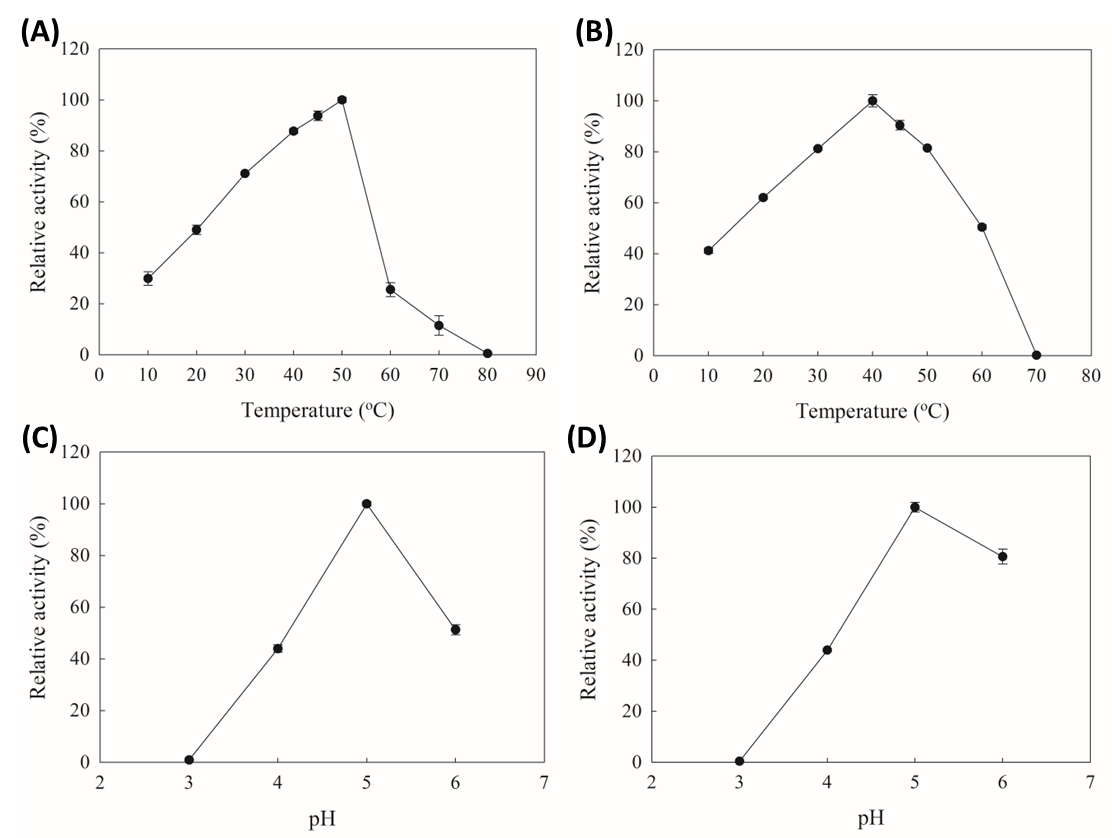


Supplementary Figure 2. Effect of temperature and pH on the activity of *Ae*GE15 (**A**, **C**) and *Tl*GE15 (**B**, **D**) on cinnamyl alcohol ester of D-glucuronic acid. All assays were carried out in duplicates.


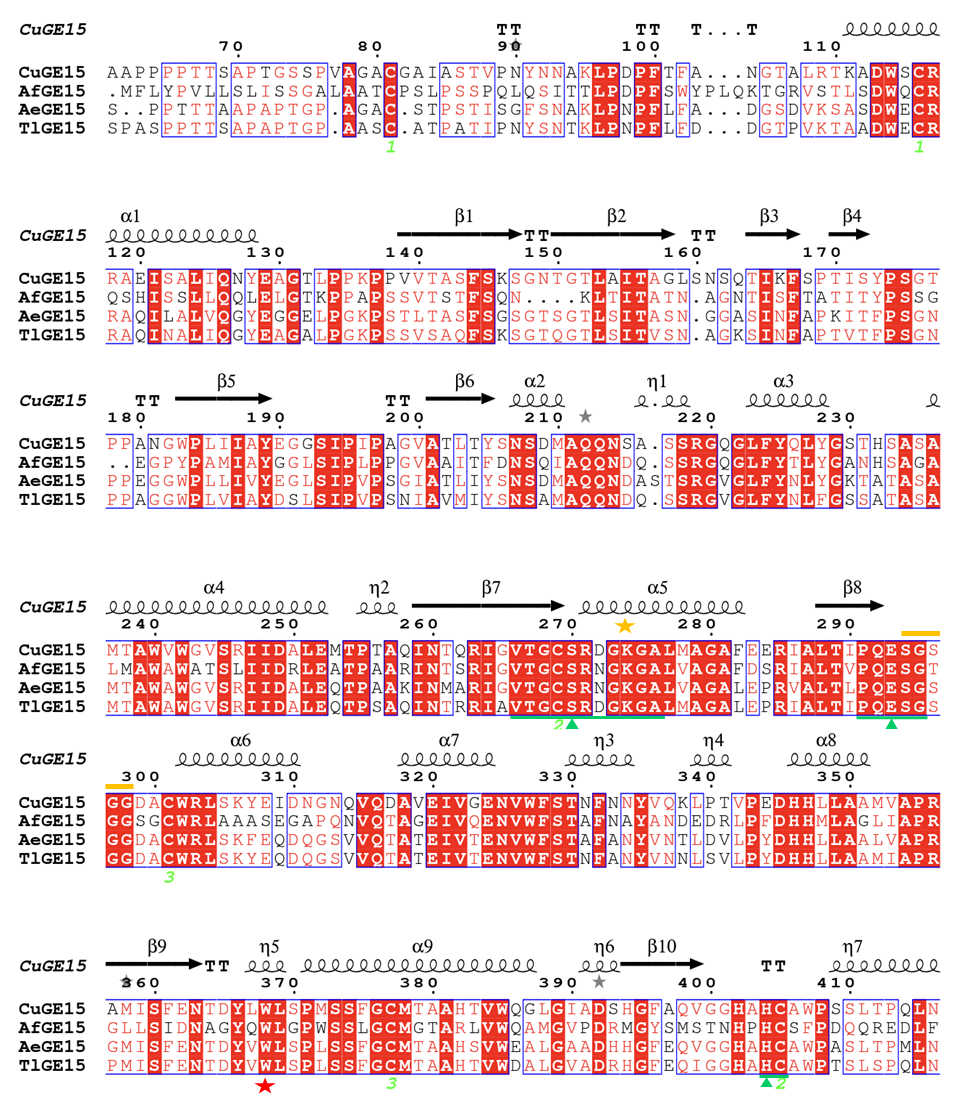


Supplementary Figure 3. Structure-based sequence alignment of *Ae*GE15, *Tl*GE15, *Af*GE15 and *Cu*GE15. Secondary structure elements are drawn as black arrows (β-sheets) and black spirals (α-helixes). The 3_10_-helixes are labeled as “η”. Strict β-turns are rendered with TT letters. Blue frames indicate similarity across groups. Identical and similar residues are printed in white on a red background and in red on a white background, respectively. The conserved motifs that include catalytic amino acids are indicated by green lines and green triangles respectively. The Lysine residue suggested to interact with the MeGlcA moiety and the “SGXGG” motif that forms the cavity for its accommodation are denoted by a yellow star and a yellow line respectively. The conserved Tryptophan of *Tt*GE15A is rendered with a red star. Green digits resemble disulfide bridges and grey stars indicate residues with alternate conformations.

Supplementary Table 1. Identity values among the amino acid sequences of *Ae*GE15 and *Tl*GE15, compared to other mature GEs from different microorganisms.

| Enzyme/  Origin | *Ae*GE15 | | *Tl*GE15 | | PDB | Reference |
| --- | --- | --- | --- | --- | --- | --- |
|  | Query coverage (%) | Percent identity (%) | Query coverage (%) | Percent identity (%) |  |  |
| *Cu*GE15/  *Cerrena unicolor* | 99 | 63.7 | 100 | 65.7 | 6RV8 | (Ernst et al., 2020) |
| *Tr*GE15/ *Trichoderma reesei* | 85 | 52.9 | 84 | 52.5 | 3PIC | (Pokkuluri et al., 2011) |
| *Lf*GE15/ *Lentithecium fluviatile* | 84 | 43.3 | 83 | 42.8 | 8B48 | (Mazurkewich et al., 2023) |
| *Tt*GE15/ *Thermothelomyces thermophilus* | 84 | 45.0 | 81 | 45.8 | 4G4G | (Charavgi et al., 2013) |

Supplementary Table 2. Different pretreatment methods of beechwood biomass and the compositional analysis of the derived samples. Data have been obtained from (Katsimpouras et al., 2017).

| A/a | Pretreatment | Acid insoluble lignin (%) | Acid soluble lignin (%) | Cellulose (%) | Hemicellulose (%) |
| --- | --- | --- | --- | --- | --- |
| 1 | Milox, 80 °C, 1 h, formic acid, 1 bar | 2.1 | 1.2 | 75.3 | 19.2 |
| 2 | distilled H_2_O/acetone (75/25 %), Air (40 % O_2_), 40 bar (175 °C, 2 h) | 11.5 | 1.0 | 84.1 | 5.2 |
| 3 | distilled H_2_O/acetone (25/75 %), Air (40 % O_2_), 40 bar (175 °C, 2 h) | 2.1 | 1.6 | 75.4 | 18.0 |
| 4 | distilled H_2_O/acetone (25/75 %), Air, 20 bar (160 °C, 2 h) | 15.7 | 3.0 | 50.2 | 19.7 |
| 5 | distilled H_2_O/acetone (50/50 %), Air (40 % O_2_), 8.5 bar (175 °C, 2 h) | 8.4 | 0.5 | 79.5 | 4.0 |
| 6 | distilled H_2_O/acetone (25/75 %), Air (40 % O_2_), 8.5 bar (175 °C, 2 h) | 13.4 | 0.7 | 73.6 | 4.2 |
| 7 | distilled H_2_O/acetone (50/50 %), Air (40 % O_2_), 40 bar (175 °C, 2 h) | 2.1 | 1.3 | 86.7 | 10.3 |
